# Comparison of exercise intensity during four early rehabilitation techniques in sedated and ventilated patients in ICU: a randomised cross-over trial

**DOI:** 10.1186/s13054-018-2030-0

**Published:** 2018-04-27

**Authors:** Clément Medrinal, Yann Combret, Guillaume Prieur, Aurora Robledo Quesada, Tristan Bonnevie, Francis Edouard Gravier, Elise Dupuis Lozeron, Eric Frenoy, Olivier Contal, Bouchra Lamia

**Affiliations:** 1 Normandie Univ, UNIROUEN, UPRES EA3830 - GRHV, Institute for Research and Innovation in Biomedicine (IRIB), 76000 Rouen, France; 2 Intensive Care Unit Department, Groupe Hospitalier du Havre, Hôpital Jacques Monod, Pierre Mendes France, 76290 Montivilliers, France; 3 Institut de Recherche Expérimentale et Clinique (IREC), Pôle de Pneumologie, ORL & Dermatologie, Université Catholique de Louvain, 1200 Brussels, Belgium; 4 Physiotherapy Department, Groupe Hospitalier du Havre, avenue Pierre Mendes France, 76290 Montivilliers, France; 5 Normandie Univ, UNIROUEN, EA3830 – GRHV, 76000 Rouen, France; 6 Institute for Research and Innovation in Biomedicine (IRIB), 76000 Rouen, France; 7 ADIR Association, Bois Guillaume, France; 8 Division of Clinical Epidemiology, Geneva University Hospitals, Geneva, Switzerland; 9 Intensive Care Unit Department Department, Groupe Hospitalier du Havre, Hôpital Jacques Monod, 76290 Montivilliers, France; 10 University of Applied Sciences and Arts Western Switzerland (HES-SO), avenue de Beaumont, 1011 Lausanne, Switzerland; 11 Intensive Care Unit, Respiratory Department, Rouen University Hospital, Rouen, France; 12 Pulmonology Department, Groupe Hospitalier du Havre, avenue Pierre Mendes France, 76290 Montivilliers, France

**Keywords:** Early rehabilitation, Intensive care unit, Mechanical ventilation, Metabolism, Sedation

## Abstract

**Background:**

In the ICU, out-of-bed rehabilitation is often delayed and in-bed exercises are generally low-intensity. Since the majority of rehabilitation is carried out in bed, it is essential to carry out the exercises that have the highest intensity. The aim of this study was to compare the physiological effects of four common types of bed exercise in intubated, sedated patients confined to bed in the ICU, in order to determine which was the most intensive.

**Methods:**

A randomised, single-blind, placebo-controlled crossover trial was carried out to evaluate the effects of four bed exercises (passive range of movements (PROM), passive cycle-ergometry, quadriceps electrical stimulation and functional electrical stimulation (FES) cycling) on cardiac output. Each exercise was carried out for ten minutes in ventilated, sedated patients. Cardiac output was recorded using cardiac Doppler ultrasound. The secondary aims were to evaluate right heart function and pulmonary and systemic artery pressures during the exercises, and the microcirculation of the vastus lateralis muscle.

**Results:**

The results were analysed in 19 patients. FES cycling was the only exercise that increased cardiac output, with a mean increase of 1 L/min (15%). There was a concomitant increase in muscle oxygen uptake, suggesting that muscle work occurred. FES cycling thus constitutes an effective early rehabilitation intervention. No muscle or systemic effects were induced by the passive techniques.

**Conclusion:**

Most bed exercises were low-intensity and induced low levels of muscle work. FES cycling was the only exercise that increased cardiac output and produced sufficient intensity of muscle work. Longer-term studies of the effect of FES cycling on functional outcomes should be carried out.

**Trial registration:**

ClinicalTrials.gov, NCT02920684. Registered on 30 September 2016.

Prospectively registered.

## Background

Early rehabilitation in intensive care has been shown to have many benefits [1]. However, the literature is inconclusive on the specific effects on muscle strength and functional capacity, with some studies showing positive effects [2–4] and others showing little effect [5]. One observational study found that despite early rehabilitation, one in two (52%) patients developed ICU-acquired weakness [6]. These contrasting results are likely due to the fact that most bed exercises are low-intensity [6]. Studies of late rehabilitation in the ICU have not found intensive rehabilitation to be more effective than standard rehabilitation [7–9]. Exercises that can be carried out early in the ICU are usually low-intensity, since the patient usually remains in bed [6, 10–12]. A study of 116 hospitals in Germany showed that only 8% of ventilated patients were taken out of bed [10]. “Out of bed” rehabilitation is limited by the presence of an endotracheal tube, sedation and confused mental state [13, 14].

There are a variety of bed exercise techniques; however, despite the fact that most rehabilitation in the ICU is carried out in bed, their effectiveness has not been evaluated. In general, the aim of exercise is to increase or maintain muscle strength and cardiovascular function. In exercise physiology, cardiac output has been shown to increase linearly from rest to maximal effort, and is one of the main responses to reflect exercise intensity [15–17]. Thus, one method of evaluating the exercise intensity is to measure cardiovascular parameters and muscle oxygenation. Theoretically, the higher the intensity of the exercise the greater is the increase in cardiac output and maximal oxygen uptake in response to the increase in muscle O_2_ consumption [15–18]. This increase in metabolism during exercise also occurs in critically ill patients [19]. To our knowledge, muscle and cardiovascular responses to different forms of bed exercise have never been compared. The most commonly used types of bed exercise are passive range of motion for the legs (PROM), passive cycle-ergometery, quadriceps electrical stimulation and functional electrical stimulation coupled with cycling (FES cycling). The main aim of this study was to compare changes in cardiac output during the four most common types of bed exercise in intubated, sedated patients confined to bed in the ICU, in order to determine which exercise had the highest intensity. The secondary aims were to evaluate right heart function and pulmonary and systemic artery pressures during the exercises and the microcirculation of the vastus lateralis muscle.

## Method

### Design

This was a randomised, controlled cross-over study carried out in an 18-bed ICU between November 2016 and July 2017. The study was approved by our institutional review board (Comité de Protection des Personnes Nord-Ouest 3). In conformity with the Declaration of Helsinki, written, informed consent to participate in the study was required from all patients. When consent was given by a proxy, the patient was informed as soon as possible and written consent was obtained. The published study protocol [20] (trial registration NCT02920684) complied with the Consolidated Standards of Reporting Trials (CONSORT) guidelines for clinical trials.

### Patients

Inclusion criteria were that patients must be over 18 years of age, intubated for at least 24 h and ventilated with “pressure support”. To avoid changes in cardiac output related to other factors such as pain, stress etc., only sedated patients with a Ramsay score greater than 4 were included. Patients were excluded if they had a pacemaker or other contraindications to electrical stimulation, if they were ventilated under “assist control ventilation”, or were conscious. Other criteria are listed in the study protocol [20].

### Procedure and randomisation

All patients participated in four consecutive 10-min sessions of bed exercise: 10 min of PROM, 10 min of quadriceps electrical stimulation, 10 min of passive cycle-ergometery (MotoMed Letto II®) and 10 min of FES cycling (RehaMove®, Hasomed, Germany). The order of the interventions was randomised using a Latin square design [20]. For the in-bed cycle-ergometry (passive peddling and FES cycling), the peddling frequency was set to 20 rev/min [2]. For the exercises involving quadriceps electrical stimulation (quadriceps electrical stimulation alone and FES-cycling), a rectangular, intermittent, bidirectional current with no ramp was used, and the intensity was modulated to obtain a palpable muscle contraction. The other electrical stimulation parameters were identical for all patients (length 300 μs, frequency 35 Hz) [20]. During FES cycling, electrical stimulation was synchronised with knee extension. A 30-min rest period was allowed between each intervention in order for the cardiorespiratory system to return to its baseline state.

### Measures

#### Primary endpoint

The primary endpoint was cardiac output during the exercises. It was measured at baseline and every 3 min during the exercises using cardiac Doppler ultrasound (CX-50®, Philips, The Netherlands) [20]. The velocity time integral (VTI) was recorded by pulsed Doppler, using an apical 5-chamber view. The VTI was computed from the area under the envelope of the pulsed-wave Doppler signal obtained at the level of the aortic annulus. The VTI was measured on an image with at least three QRS complexes on the electrocardiogram. A mean of 3 VTIs was calculated every 3 min. Cardiac output was calculated using the formula: VTI × CSA × cardiac frequency, where CSA represents cross-sectional area. All measurements were carried out by a technician with two years’ experience of ultrasound in the ICU, and were analysed by an expert doctor.

#### Secondary endpoints

Tricuspid annular plane systolic excursion (TAPSE) using an apical 4-chamber view, pulmonary arterial systolic pressure (PASP) (the sum of the pressure gradient between the right ventricle and the pulmonary artery and right atrial pressure), mean arterial pressure (MAP), expiratory tidal volume and respiratory rate were measured at baseline and every 3 min during the exercises. The mean value during the exercise was calculated for each parameter.

Relative change in total haemoglobin in the vastus lateralis muscle (THb), oxyhaemoglobin and oxymyoglobin (HbO_2_) and deoxyhaemoglobin and deoxymyoglobin (HHb) were continuously recorded using a wireless, portable, near infrared spectroscopy (NIRS) device (Portamon, Artinis, The Netherlands). The NIRS optode was positioned longitudinally 10 cm above the patella on the right vastus lateralis and was continuously recorded at a sampling frequency of 1 Hz. The optode was fixed to the skin with a black band to avoid the influence of room light.

### Statistical analysis

A power calculation showed that 19 subjects should be included to detect a difference between groups in mean CO of 1.1 L, and to reject the null hypothesis with power of 90% and associated type I probability error of 0.05. It was thus planned to include 20 patients in total [20]. Descriptive statistics are reported as counts and percentages for categorical data and means and standard deviations or medians and interquartile ranges for continuous variables. To compare the effect of the different exercises on the primary endpoint across the different measurement times (baseline, 3 min, 6 min and 9 min), a linear mixed effects model with a random intercept was used for each participant, and interactions between exercise type and measurement time were analysed. The same model was used to compare the effect of the interventions on THb, HbO_2_ and HHb; however, baseline measures were not included since they were zero in all patients. For the other secondary endpoints, the effect of the interventions on the difference between baseline measurements and mean values during the exercises was also assessed using a linear mixed model with a random intercept for each participant and analysis of interactions between exercise type and measurement time. All models were adjusted for the intervention sequence. The statistical significance of the interactions was assessed using the likelihood ratio test. All analyses were performed using R version 3.4.2 and lme4 and lsmeans software. A two-tailed *p* value of 0.05 was considered significant.

## Results

**Fig. 1:**
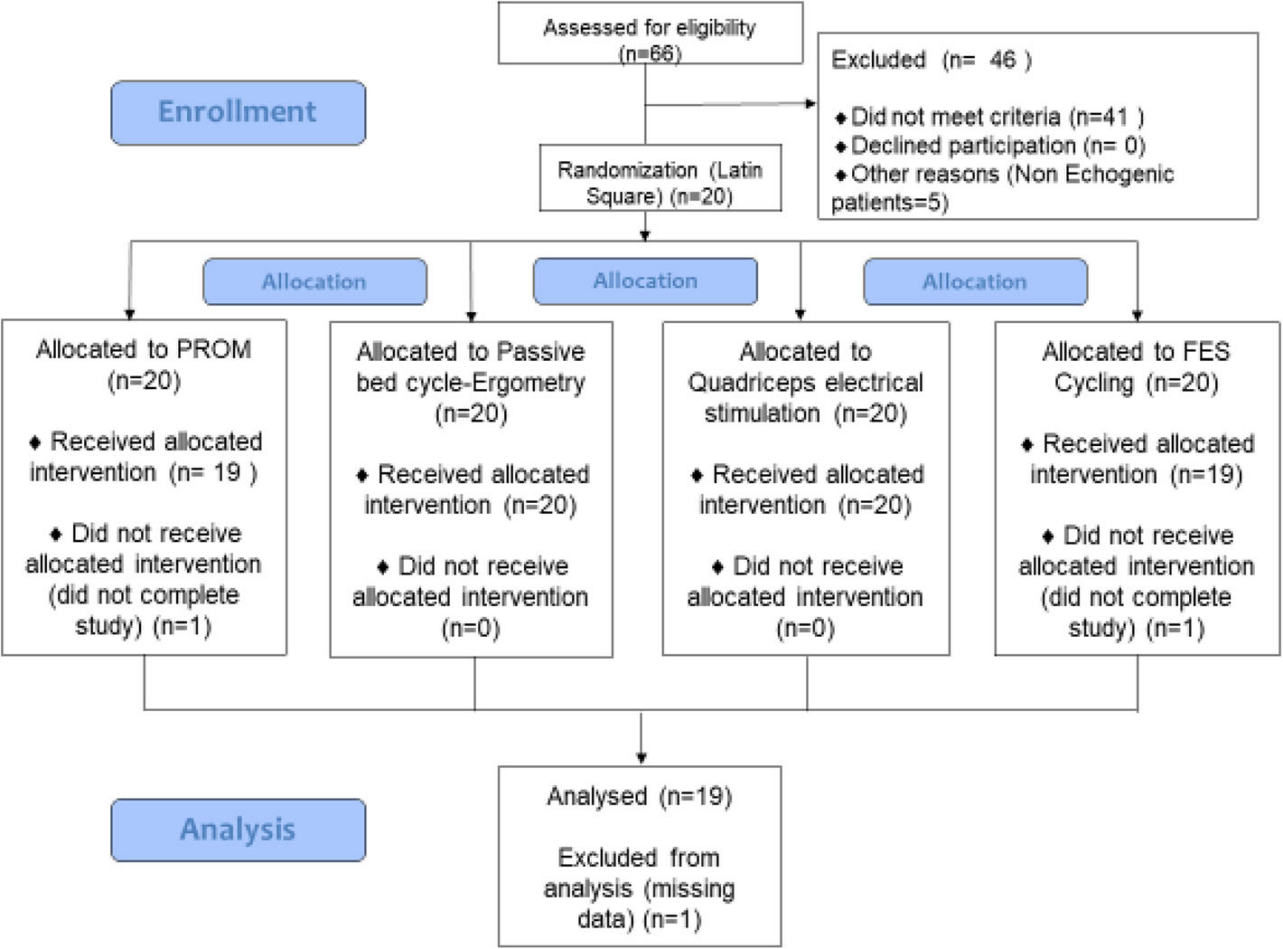
Study design. PROM, passive range of leg movement; FES, functional electrical stimulation

Twenty patients were included. One patient was excluded from the analysis because only two of the four interventions were carried out because the ultrasound machine was needed for an emergency in another patient (See Fig. 1). Patient characteristics are summarised in Table 1. Briefly, 32% were female, mean age was 63 years and median body mass index (BMI) was 29.7 kg/m^2^. The mean Ramsay score was 6/6 and mean duration of ventilation at inclusion was 4 days. Four patients were undergoing renal dialysis and four were receiving vasoactive drugs (at a dose < 0.5 μg/kg/min).

**Table 1:** Patient characteristics

Characteristics	Value (*N* = 19 patients)
Female, *n* (%)	6 (32)
Age, mean (SD)	65.3 (9.7)
Body mass index (kg/m^2^), median (25–75th percentile)	29.7 (22.5–32.7)
SAPS II at ICU admission, mean (SD)	57.5 (24)
Main diagnosis	
Pneumonia, *n* (%)	4 (21)
Sepsis, *n* (%)	1 (5)
COPD/asthma exacerbation, *n* (%)	1 (5)
Cardiac failure, *n* (%)	3 (16)
Drug overdose/acute mental status change, *n* (%)	5 (26)
Intra-abdominal sepsis with surgery, *n* (%)	5 (26)
Co-morbidity	
Chronic pulmonary disease, *n* (%)	6 (32)
Obesity, *n* (%)	9 (47)
Chronic cardiac insufficiency, *n* (%)	5 (26)
Cancer, *n* (%)	1 (5)
Chronic kidney disease, *n* (%)	3 (16)
Diabetes mellitus, *n* (%)	7 (37)
Between admission and inclusion	
Septic shock, *n* (%)	6 (31)
ARDS, *n* (%)	5 (26)
Renal failure, *n* (%)	8 (42)
Use of cathecolamines, *n* (%)	13 (68)
Use of neuromuscular blockers, *n* (%)	10 (53)
No. of days of neuromuscular blockers, median (25–75th percentile)	1 (0–2)
Ventilator use (days), median (25–75th percentile)	4 (2–7)
Ventilator parameters and sedation use during protocol	
Pressure support (cmH_2_O), mean (SD)	15 (3)
Positive end-expiratory pressure (cmH_2_O), mean (SD)	7 (1)
Fraction of inspired oxygen (%), mean (SD)	35 (13)
Midazolam mg/h, mean (SD)	5 (4)

*SAPS* Simplified Acute physiology Score, *ICU* intensive care unit, *n* number, *COPD* chronic obstructive pulmonary disease, *ARDS* acute respiratory distress syndrome

### Primary outcome

**Fig. 2:**
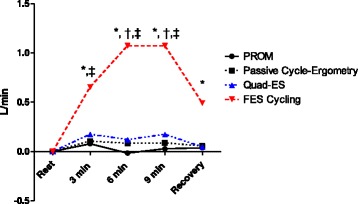
Cardiac output over time for each exercise. Black circles represent passive range of leg movement (PROM); black squares represent passive cycle-ergometry; blue triangles represent quadriceps electrical stimulation; red triangles represent functional electrical stimulation cycling (FES-Cycling). *Significantly different between PROM and FES-Cycling; ^†^significantly different between passive cycle-ergometery and FES-Cycling; ^‡^significantly different between quadriceps electrical stimulation and FES-Cycling

There were no differences in cardiac output at rest before each exercise (see Table 2). Figure 2 shows cardiac output over time. Cardiac output increased significantly (+ 1 L/min) after 9 min of FES cycling. There was no change in cardiac output over time during PROM, passive cycle ergometry or quadriceps electrical stimulation. There were no differences between the increase in cardiac output during FES cycling in patients with or without cardiorespiratory comorbidities (chronic obstructive pulmonary disease (COPD) or chronic heart failure).

**Table 2:** Cardiac ouput values during the four types of bed exercise

Cardiac output (L/min) (95% CI)	Passive range of leg motion (PROM)	Passive cycle-ergometery	Quadriceps electrical stimulation	Functional electrical stimulation cycling (FES cycling)
Rest	6.6 (5.6–7.3)	6.7 (5.8–7.7)	6.6 (5.7–7.6)	6.7 (5.7–7.6)
3 min	6.6 (5.6–7.5)	6.8 (5.8–7.8)	6.8 (5.8–7.7)	7.3 (6.3–8.3)^*, ‡^
6 min	6.5 (5.5–7.5)	6.8 (5.8–7.7)	6.7 (5.8–7.7)	7.7 (6.8–8.7)^*, †,‡^
9 min	6.5 (5.6–7.5)	6.8 (5.8–7.7)	6.8 (5.8–7.7)	7.7 (6.7–8.7)^*, †,‡^
Recovery	6.6 (5.6–7.5)	6.7 (5.7–7.6)	6.6 (5.7–7.6)	7.1 (6.2–8.1)^*^

*Significant difference between PROM and FES cycling; †significant difference between passive cycle-ergometery and FES cycling; ^‡^significant difference between quadriceps electrical stimulation and FES cycling

### Secondary outcomes

There were no differences between the secondary outcomes at rest before each exercise. There was a significant increase in heart rate, TAPSE and MAP during FES cycling (see Table 3). MAP also increased during passive cycle ergometry. Cardiorespiratory parameters did not change during the other exercises.

**Table 3:** Secondary outcomes at rest and during exercise

Outcomes	PROM		Passive cycle ergometry		Quadriceps electrical stimulation		FES cycling	
	Rest	Exercise	Rest	Exercise	Rest	Exercise	Rest	Exercise
Heart rate (b/min)	93 (86–100)	93 (86–100)	94 (86–101)	94 (86–101)	94 (86–101)	93 (86–101)	94 (86–101)	97^*^ (90–104)
TAPSE (cm)	2 (1.8–2.2)	1.9 (1.7–2.1)	1.8 (1.5–2)	1.8 (1.6–2)	1.7 (1.5–2)	1.8 (1.6–2)	1.8 (1.6–2)	2^*^ (1.8–2.2)
Mean arterial pressure (mmHg)	87 (80–93)	88 (82–94)	85 (79–91)	89^*^ (83–95)	87 (80–93)	87 (81–93)	84 (77–90)	91^*^ (85–97)
PAPS (mmHg)	51 (37–66)	45 (32–59)	51 (39–63)	49 (35–62)	47 (35–58)	46 (35–57)	50 (36–64)	51^†,‡^ (36–67)
Respiratory Rate (c/min)	20 (16–24)	20 (16–24)	22 (17–27)	22 (17–27)	20 (15–25)	21 (16–26)	21 (17–25)	24^†,‡^ (19–30)
Tidal volume (mL)	513 (447–579)	507 (443–571)	514 (427–600)	527 (449–605)	521 (446–596)	497 (441–553)	510 (427–593)	521 (446–596)

*PROM* passive range of motion, *TAPSE* tricuspid annular plane systolic excursion, *PAPS* pulmonary arterial systolic pressure

*Significant difference between rest and exercise; †significant difference between PROM and FES cycling during exercise; ‡significant difference between quadriceps electrical stimulation and FES cycling

PASP was significantly higher during FES cycling than PROM and quadriceps electrical stimulation (respectively, 51 (95% CI 36–67) mmHg vs. 45 (95% CI 32–59) mmHg (*p* = 0.007) vs. 46 (95% CI 35–57) mmHg (*p* < 0.001)).

Respiratory rate was significantly higher during FES cycling than during PROM and quadriceps electrical stimulation (respectively, 24 (95% CI 19–30) c/min vs. 20 (95% CI 16–24) c/min (*p* < 0.001) vs. 21 (95% CI 16–26) c/min (*p* = 0.005)).

**Fig. 3:**
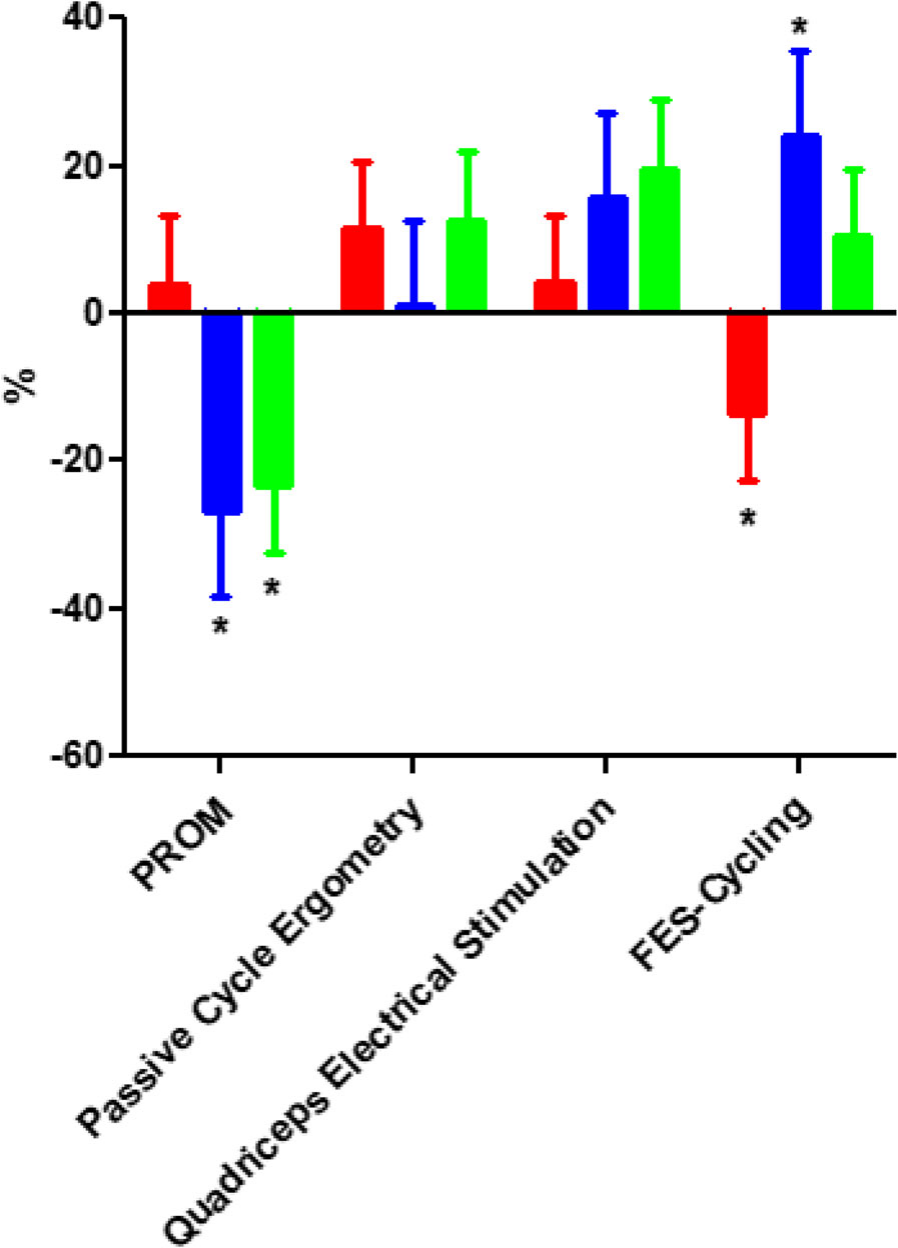
Relative change in haemoglobin at the end of each exercise. Red bars represent oxyhaemoglobin (HbO_2_); blue bars represent deoxyhaemoglobin (HHb); green bars represent total haemoglobin (THb); *p < 0.05 for comparison between baseline and the end of the exercise

At the end of the PROM, the level of THb had decreased significantly by 23% (95% CI − 41.5 to − 4.9) (*p* = 0.046). This led to a significant reduction in HHb level (− 27% (95% CI − 50 to − 4) (See Fig. 3). HbO_2_ did not change.

At the end of the passive cycle-ergometry, there was a non-significant increase in THb (+ 12.7% (95% CI − 5.6 to 31) (*p* = 0.3) compared with rest, with an associated non-significant increase in HbO_2_ (+ 11% (95% CI − 7 to 30).

There was a non-significant increase in THb (+ 19.5% (95% CI − 1.1 to 37.8) (*p* = 0.08) at the end of the quadriceps electrical stimulation. This produced a non-significant increase in HHb (+ 15.5% (95% CI − 7.2 to 38.2). There was no change in HbO_2_. The non-significant increase in THb (+ 10.3% (95% CI − 8 to 28.6) (*p* = 0.3) during FES cycling was induced by a significant increase in HHb of 24% (95% CI 1.1–46.7). HbO_2_ decreased significantly by 13% (95% CI − 31.8 to − 4.7). No adverse events occurred during the exercises.

## Discussion

To our knowledge, this is the first study to compare different types of bed exercise in the ICU. The present study showed that (1) most bed exercises were low-intensity and induced a low level of muscle work and (2) only FES cycling increased cardiac output and physiological cardiorespiratory response and reduced muscle HbO_2_.

### Cardiorespiratory response to exercise

The principle of physical exercise is to increase muscle work in order to increase metabolic energy demand. The increase in muscle activity leads to an increase in metabolic consumption of O_2_ and cardiac output in order to maintain muscle perfusion and sufficient levels of O_2_ [16–18]. Of the four interventions, only FES cycling increased cardiac output. The increase was considerable, around 15% (1 L/min). The slight increase in cardiac frequency suggests that stroke volume increased, thus demonstrating a global increase in cardiac activity [21]. This increase was clinically significant and showed that FES cycling produced a cardiac response to the exercise, in contrast with the other interventions that did not modify cardiac activity. Moreover, the other cardiovascular and respiratory parameters also increased with FES cycling. Although this may be difficult to interpret clinically, based on principles from applied physiology, the increase in TAPSE, MAP, PASP and respiratory rate confirms our hypothesis of an increase in cardiac activity and exercise intensity [15, 16, 22, 23].

Few physiological data are available in the literature on early rehabilitation in the ICU. The majority of studies simply report cardiac frequency or SpO_2_ [24, 25]. Moreover, FES cycling is a relatively new technique and therefore few studies have evaluated its use in the ICU [26]. Muraki et al. observed an increase in cardiac output and oxygen consumption (VO_2_) during FES cycling in patients with American Spinal Injury Association (ASIA) A- paraplegia, with no changes during passive leg cycling [27]. A study in critically ill patients observed a 1% increase in cardiac output with passive leg cycling in sedated patients [28]. These results are in accordance with the present study, which showed that neither of the passive techniques induced a cardiac response. Moreover, there is little rationale to support the use of passive exercises to improve muscle function [29]. However, the results of the present study also showed that quadriceps electrical stimulation did not increase cardiac output. Although several studies have reported the benefits of neuromuscular electrical stimulation in the ICU [4, 30], other studies have found no benefits and further studies are necessary to determine the true effects of this intervention [31]. It is important to note that quadriceps electrical stimulation can prevent muscle atrophy by improving glucose metabolism [30, 32, 33].

### Effects on muscle

Changes in the microcirculation in the vastus lateralis muscle during the interventions were evaluated using NIRS. THb reflects the blood volume of the tissue under the probe, and it is assumed that HHb reflects muscle O_2_ extraction [34, 35]. During the PROM, muscle blood volume reduced, probably as a result of the vertical position of the femur during the flexion movements, as has been described previously in passive leg raising [36]. The lack of a muscle strengthening effect was reinforced by the decrease in HHb during the intervention. Conversely, during passive leg cycling, blood volume did not decrease significantly. Although the position of the limb was similar to that during the PROM, we believe that the regular rhythm of the passive cycling (20 rotations/min) altered the circulation in the lower limb [37]. However, the lack of change in HHb demonstrates that the passive cycle-ergometery did not effectively increase muscle metabolism.

During the quadriceps electrical stimulation, HHb increased (although not significantly), suggesting an increase in muscle metabolism. This is in accordance with the literature that shows that neuromuscular stimulation has an effect on local metabolism, but not on the cardiovascular system [38, 39]. This lack of effect on cardiac output probably explains why several studies have failed to show changes in functional capacity following quadriceps electrical stimulation [31].

During the FES cycling, there was a significant reduction in HbO_2,_ and a significant increase in HHb, despite the increase in cardiac output. This demonstrates that this exercise induced muscle consumption of O_2_ and increased muscle metabolism [40].

### Clinical implications

In our clinical practice, we try to use exercises that involve voluntary movement, in preference out of bed. However, many studies have shown that a large proportion of rehabilitation in our ICU is carried out in bed [6, 10–12], and this is also the case in our ICU. The results of this study suggest interventions that induce muscle contractions, particularly FES cycling, should be used to provide higher-intensity early rehabilitation for sedated patients who are confined to bed. Passive interventions can be used to prevent ankylosis but studies are needed to evaluate their effectiveness.

### Study limitations

This study has several limitations. The main limitation is that the long-term effects on muscle parameters, such as muscle fibre atrophy, and short and long-term functional capacity were not evaluated. Furthermore, it is important to bear in mind that an increase in exercise intensity may not improve the patient’s prognosis. Although our results showed differences in exercise metabolism between the four types of early rehabilitation exercises, the study was not designed to evaluate functional capacity. However, the results provide a strong physiological rationale for the use of interventions that combine muscle contractions and movement therapy. We chose to evaluate cardiac output based on the linear relationship between this parameter and exercise intensity [15–17], however, cardiac output does not actually quantify the intensity of the exercise. The exercise response is also dependent on the oxidation of macronutrients, and a measurement of energy expenditure using calorimetry could have precisely quantified the exercise intensity. The second limitation is the fact that only sedated patients who were confined to bed were included, thus the results can only be generalised to this specific population. Nevertheless, positioning and exercising out of bed have already been shown to be effective, while there is a lack of studies of the benefits of in-bed rehabilitation. Moreover, it is these patients who are the most at risk of developing ICU-acquired weakness [41, 42]. We chose to evaluate sedated patients to avoid the effects of confounding factors, such as stress or discomfort, on cardiac output. Another limitation is the lack of statistical power for the evaluation of changes in muscle metabolism, which probably resulted in the lack of significance in the NIRS parameters. Moreover, NIRS only provides an estimation of the parameters evaluated and its application is limited to 3–4 cm below the probe [43]. However, it provided complementary information that was useful for the interpretation of the cardiovascular results.

## Conclusion

Evaluation of the main interventions used for early rehabilitation in the ICU showed that only FES cycling increased cardiac output and produced sufficient intensity of muscle work to constitute an effective early rehabilitation intervention. No muscle or systemic effects were induced by the passive techniques. Longer-term studies of the effect of FES cycling for preventing muscle atrophy or improving functional outcomes should be carried out.

## Abbreviations

CO: Cardiac output
FES: Functional electrical stimulation
HbO2: Oxyhaemoglobin
HHb: Deoxyhaemoglobin
MAP: Mean arterial pressure
MV: Mechanical ventilation
NIRS: Near infrared spectrospcopy
PASP: Pulmonary arterial systolic pressure
PROM: Passive range of leg movement
TAPSE: Tricuspid annular plane systolic excursion
THb: Total haemoglobin

## References

[1] Denehy L, Lanphere J, Needham DM (2017). Ten reasons why ICU patients should be mobilized early. Intensive Care Med.

[2] Burtin C, Clerckx B, Robbeets C (2009). Early exercise in critically ill patients enhances short-term functional recovery. Crit Care Med.

[3] Schweickert WD, Pohlman MC, Pohlman AS (2009). Early physical and occupational therapy in mechanically ventilated, critically ill patients: a randomised controlled trial. Lancet.

[4] Routsi C, Gerovasili V, Vasileiadis I (2010). Electrical muscle stimulation prevents critical illness polyneuromyopathy: a randomized parallel intervention trial. Crit Care.

[5] Castro-Avila AC, Seron P, Fan E (2015). Effect of Early Rehabilitation during Intensive Care Unit Stay on Functional Status: Systematic Review and Meta-Analysis. PLoS One.

[6] Hodgson C, Bellomo R, Berney S (2015). Early mobilization and recovery in mechanically ventilated patients in the ICU: a bi-national, multi-centre, prospective cohort study. Crit Care.

[7] Morris PE, Berry MJ, Files DC (2016). Standardized rehabilitation and hospital length of stay among patients with acute respiratory failure: a randomized clinical trial. JAMA.

[8] Moss M, Nordon-Craft A, Malone D (2016). A randomized trial of an intensive physical therapy program for patients with acute respiratory failure. Am J Respir Crit Care Med.

[9] Wright SE, Thomas K, Watson G (2017). Intensive versus standard physical rehabilitation therapy in the critically ill (EPICC): a multicentre, parallel-group, randomised controlled trial. Thorax..

[10] Nydahl P, Ruhl AP, Bartoszek G (2014). Early mobilization of mechanically ventilated patients: a 1-day point-prevalence study in Germany. Crit Care Med.

[11] Connolly BA, Mortimore JL, Douiri A, et al. Low levels of physical activity during critical illness and weaning: the evidence-reality gap. J Intensive Care Med. 2017:885066617716377. 10.1177/0885066617716377 PMC6716208 28675113

[12] Berney SC, Rose JW, Bernhardt J (2015). Prospective observation of physical activity in critically ill patients who were intubated for more than 48 hours. J Crit Care.

[13] Jolley SE, Moss M, Needham DM (2017). Point prevalence study of mobilization practices for acute respiratory failure patients in the United States. Crit Care Med.

[14] Parry SM, Knight LD, Connolly B (2017). Factors influencing physical activity and rehabilitation in survivors of critical illness: a systematic review of quantitative and qualitative studies. Intensive Care Med.

[15] Forton K, Motoji Y, Deboeck G (2016). Effects of body position on exercise capacity and pulmonary vascular pressure-flow relationships. J Appl Physiol (1985).

[16] Kovacs G, Herve P, Barbera JA, et al. An official European Respiratory Society statement: pulmonary haemodynamics during exercise. Eur Respir J. 2017;50(5) 10.1183/13993003.00578-2017 29167297

[17] Trinity JD, Lee JF, Pahnke MD (2012). Attenuated relationship between cardiac output and oxygen uptake during high-intensity exercise. Acta Physiol (Oxford).

[18] Gormley SE, Swain DP, High R (2008). Effect of intensity of aerobic training on VO2max. Med Sci Sports Exerc.

[19] Hickmann CE, Roeseler J, Castanares-Zapatero D (2014). Energy expenditure in the critically ill performing early physical therapy. Intensive Care Med.

[20] Medrinal C, Combret Y, Prieur G (2017). Effects of different early rehabilitation techniques on haemodynamic and metabolic parameters in sedated patients: protocol for a randomised, single-bind, cross-over trial. BMJ Open Respir Res.

[21] Lamia B, Ochagavia A, Monnet X (2007). Echocardiographic prediction of volume responsiveness in critically ill patients with spontaneously breathing activity. Intensive Care Med.

[22] Lamia B, Teboul JL, Monnet X (2007). Relationship between the tricuspid annular plane systolic excursion and right and left ventricular function in critically ill patients. Intensive Care Med.

[23] Naeije R, Saggar R, Badesch D, et al. Exercise-Induced pulmonary hypertension: translating pathophysiological concepts into clinical practice. Chest. 2018;(18):30161–2. 10.1016/j.chest.2018.01.022 29382472

[24] Bailey P, Thomsen GE, Spuhler VJ (2007). Early activity is feasible and safe in respiratory failure patients. Crit Care Med.

[25] Bourdin G, Barbier J, Burle JF (2010). The feasibility of early physical activity in intensive care unit patients: a prospective observational one-center study. Respir Care.

[26] Parry SM, Berney S, Warrillow S (2014). Functional electrical stimulation with cycling in the critically ill: a pilot case-matched control study. J Crit Care.

[27] Muraki S, Fornusek C, Raymond J (2007). Muscle oxygenation during prolonged electrical stimulation-evoked cycling in paraplegics. Appl Physiol Nutr Metab.

[28] Camargo Pires-Neto R, Fogaca Kawaguchi YM, Sayuri Hirota A (2013). Very early passive cycling exercise in mechanically ventilated critically ill patients: physiological and safety aspects–a case series. PLoS One.

[29] Hodgson CL, Berney S, Harrold M (2013). Clinical review: early patient mobilization in the ICU. Crit Care.

[30] Gerovasili V, Stefanidis K, Vitzilaios K (2009). Electrical muscle stimulation preserves the muscle mass of critically ill patients: a randomized study. Crit Care.

[31] Parry SM, Berney S, Granger CL (2013). Electrical muscle stimulation in the intensive care setting: a systematic review. Crit Care Med.

[32] Gruther W, Kainberger F, Fialka-Moser V (2010). Effects of neuromuscular electrical stimulation on muscle layer thickness of knee extensor muscles in intensive care unit patients: a pilot study. J Rehabil Med.

[33] Weber-Carstens S, Schneider J, Wollersheim T (2013). Critical illness myopathy and GLUT4-significance of insulin and muscle contraction. Am J Respir Crit Care Med.

[34] Ferreira LF, Koga S, Barstow TJ (2007). Dynamics of noninvasively estimated microvascular O2 extraction during ramp exercise. J Appl Physiol.

[35] Van Beekvelt MC, Colier WN, Wevers RA (2001). Performance of near-infrared spectroscopy in measuring local O(2) consumption and blood flow in skeletal muscle. J Appl Physiol.

[36] Monnet X, Marik P, Teboul JL (2016). Passive leg raising for predicting fluid responsiveness: a systematic review and meta-analysis. Intensive Care Med.

[37] Nobrega AC, Williamson JW, Friedman DB (1994). Cardiovascular responses to active and passive cycling movements. Med Sci Sports Exerc.

[38] Gerovasili V, Tripodaki E, Karatzanos E (2009). Short-term systemic effect of electrical muscle stimulation in critically ill patients. Chest.

[39] Angelopoulos E, Karatzanos E, Dimopoulos S (2013). Acute microcirculatory effects of medium frequency versus high frequency neuromuscular electrical stimulation in critically ill patients - a pilot study. Ann Intensive Care.

[40] Subudhi AW, Dimmen AC (2007). Roach RC. Effects of acute hypoxia on cerebral and muscle oxygenation during incremental exercise. J Appl Physiol.

[41] Fan E, Cheek F, Chlan L (2014). An official American Thoracic Society Clinical Practice guideline: the diagnosis of intensive care unit-acquired weakness in adults. Am J Respir Crit Care Med.

[42] Latronico N, Herridge M, Hopkins RO (2017). The ICM research agenda on intensive care unit-acquired weakness. Intensive Care Med..

[43] Neary JP (2004). Application of near infrared spectroscopy to exercise sports science. Can J Appl Physiol.

